# Supplementary material for: Bei Mu Gua Lou San facilitates mucus expectoration by increasing surface area and hydration levels of airway mucus in an air-liquid-interface cell culture model of the respiratory epithelium
Source: BMC Complement Med Ther. 2023 Nov 17;23:414. doi: 10.1186/s12906-023-04251-x (PMC10655387; doi:10.1186/s12906-023-04251-x)
Supplement: Supplementary file 1 — Supplementary Material 1 [file 12906_2023_4251_MOESM1_ESM.pdf]

Supplementary Information file

Characterization of NHBE cells after isolation

We confirmed the viability/ quality of isolated NHBE cells immediately after isolation by trypan blue staining and Casy Cell Counter measurements. To check identity of cells we used IF to verify individual cell types in the re-established pseudostratified epithelium. αTubulin (αTub) was used to target apical cilia while MUC5AC and uteroglobin (CCSP) mark the presence of goblet and Club cells, respectively. Cilia formation was present consistently in NHBE ALI cultures after 3 weeks of differentiation similarly to MUC5AC (representative images shown in Supplemental Figure 1 a, b). Interestingly, CCSP was detected only scarcely in normal NHBE ALI cultures (Supplemental Figure 1 a, b; white arrows).

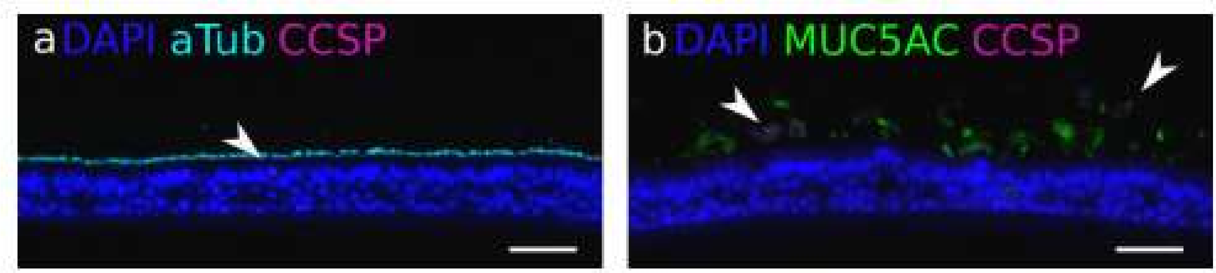

Supplemental Figure 1. Expression of αTub, CCSP and MUC5AC in ALI cultures. Representative images of (a) αTub that was expressed comprehensively across the sample and (b) MUC5AC and CCSP, marking the presence of goblet and club cells, respectively. CCSP was present only in sparse amounts (a, b; arrowheads). Scale bars, 20 µm.

Full-length western blot of ALOX15 and beta ACT

We tested interference of BMGLS with the major mucus-stimulating enzyme ALOX15 and found diminished levels of ALOX15 in BMGLS treated samples (Supplemental Figure 2).

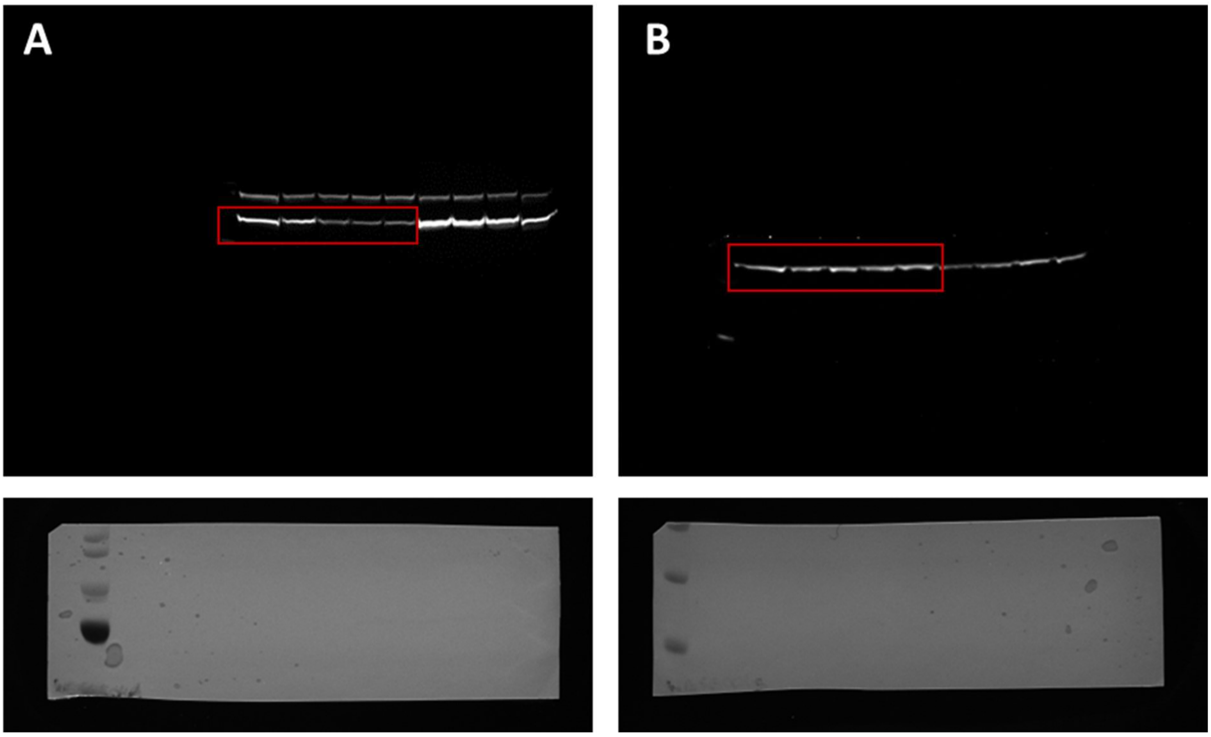

Supplemental Figure 2. Protein expression of ALOX15 in NHBE ALI cultures treated with BMGLS for 3 weeks. ALOX15 (A) and beta ACT (B) were shown in two individual western blots with exposure time of 280ms.

Representative IF images of BMGLS treated Calu-3 ALI cultures

Calu-3 ALI cultures were treated with different concentrations of BMGLS over the course of 3 weeks. Representative IF image of proliferating cells (Ki67, Cy3), apoptotic cells (cPARP, Cy5) and nuclei (DAPI). Magnification 20x.

Calu-3 Cntrl Cy3	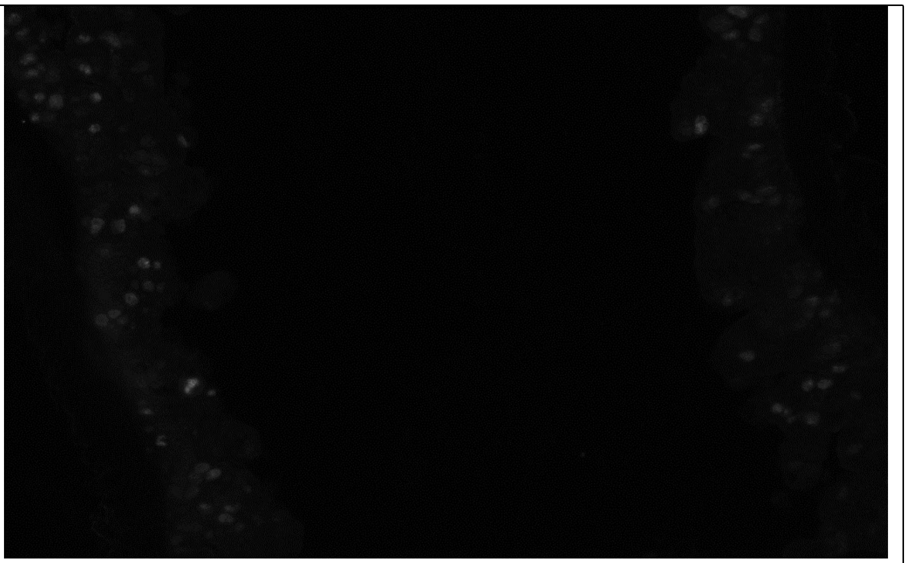
Calu-3 Cntrl Cy5	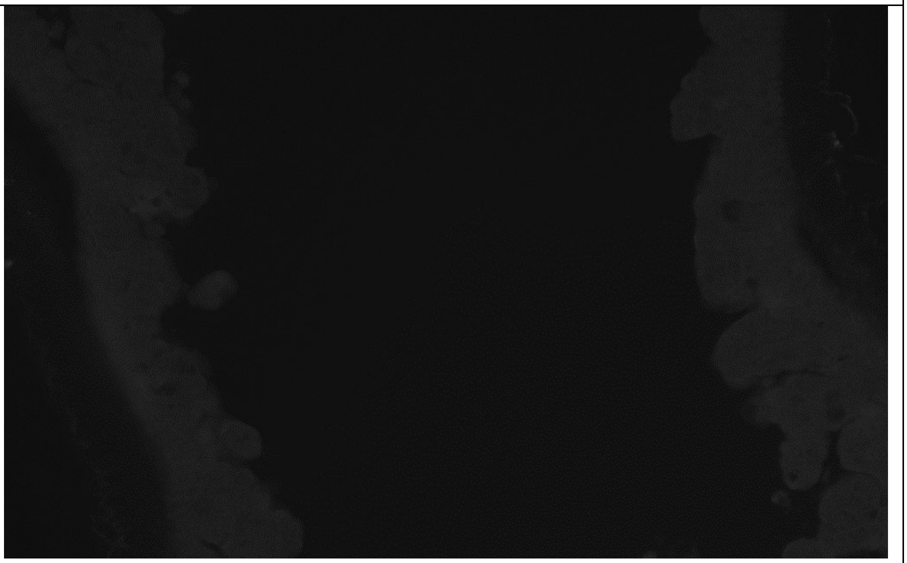

Calu-3 Cntrl DAPI	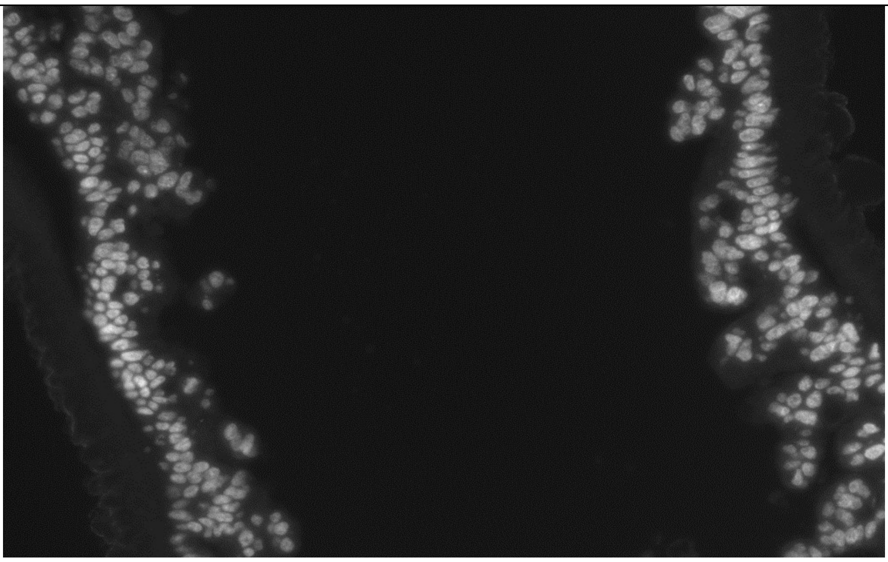

Calu-3 HCS Cy3	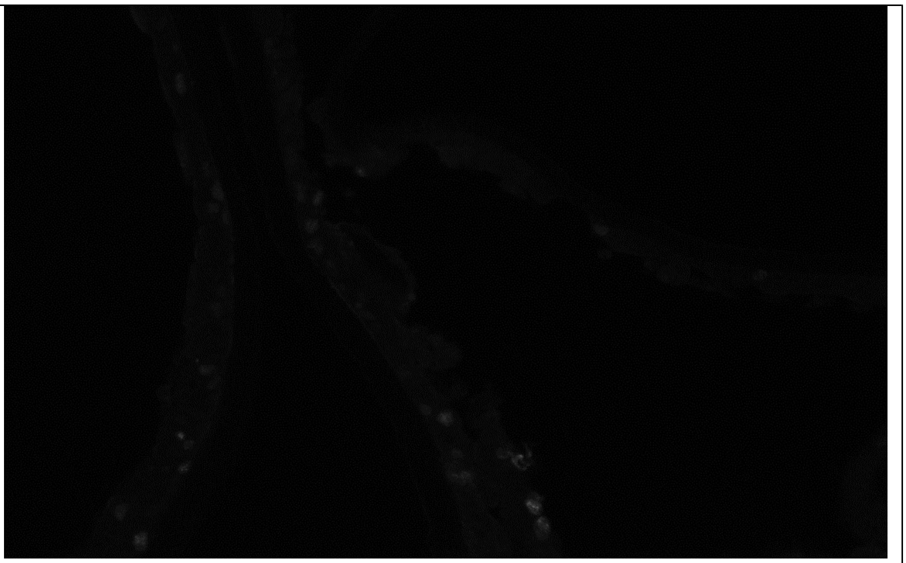
Calu-3 HCS Cy5	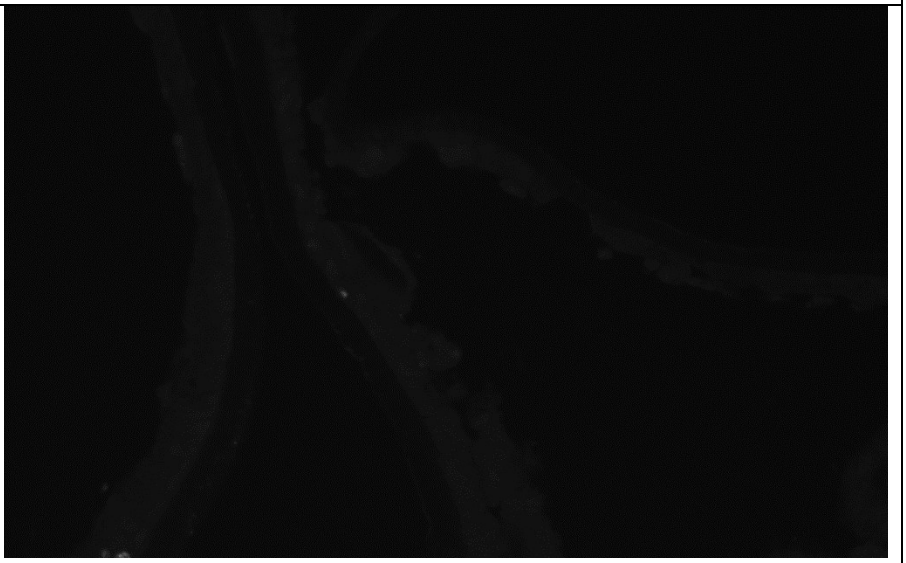

Calu-3 HCS DAPI	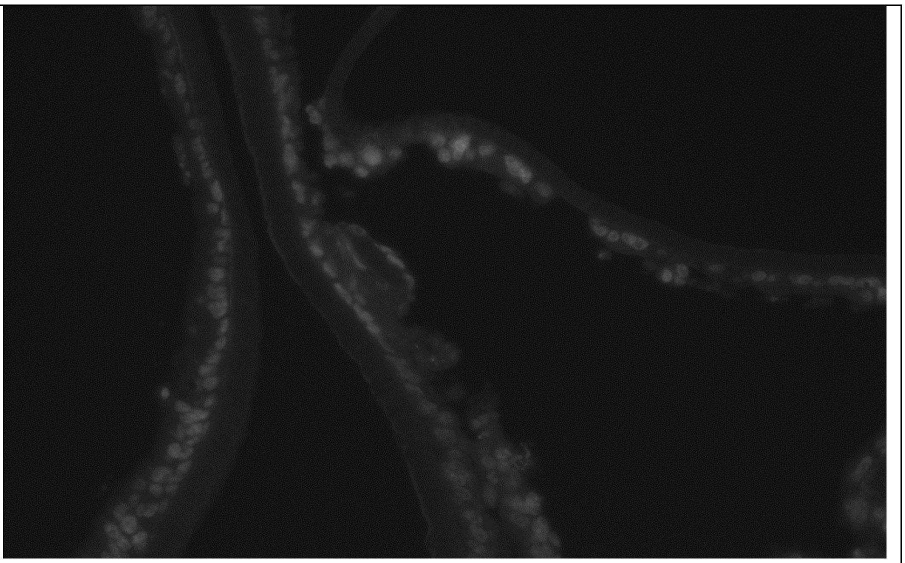

Calu-3 BMGLS 0.15% Cy3	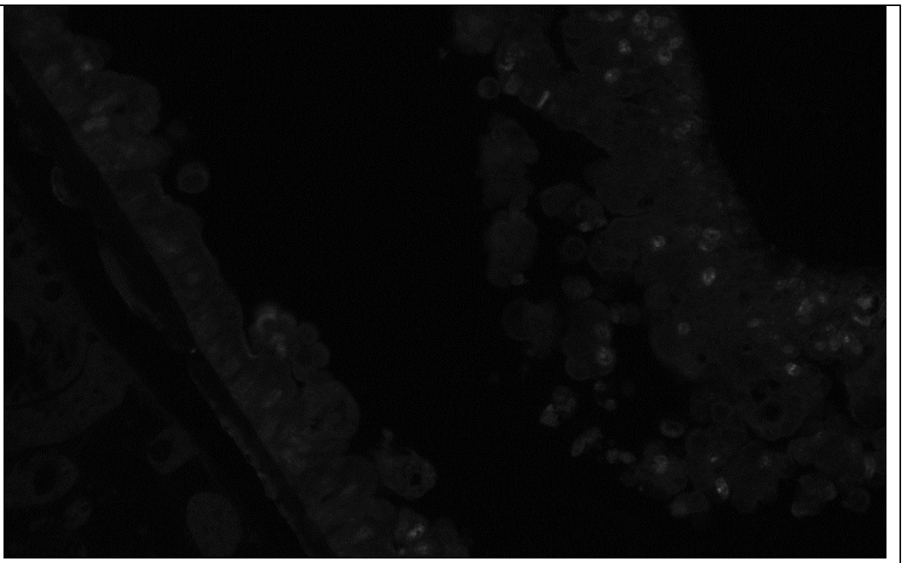

Calu-3 BMGLS 0.15% Cy5	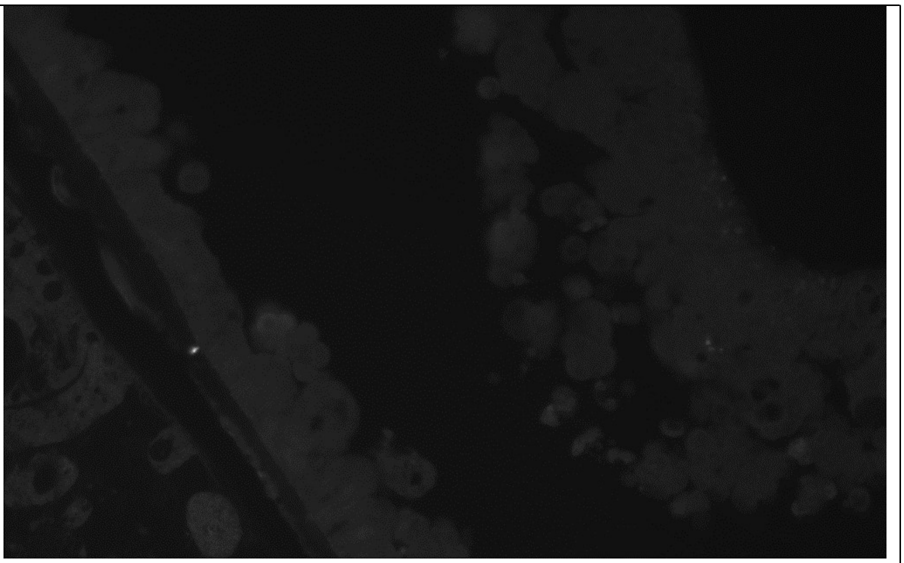
Calu-3 BMGLS 0.15% DAPI	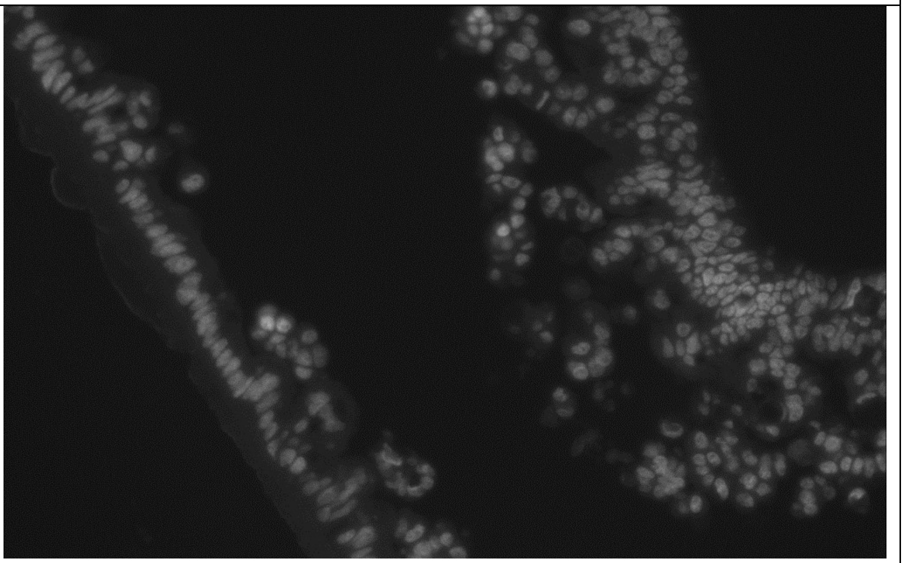

Calu-3 BMGLS 0.3%<br>Cy3	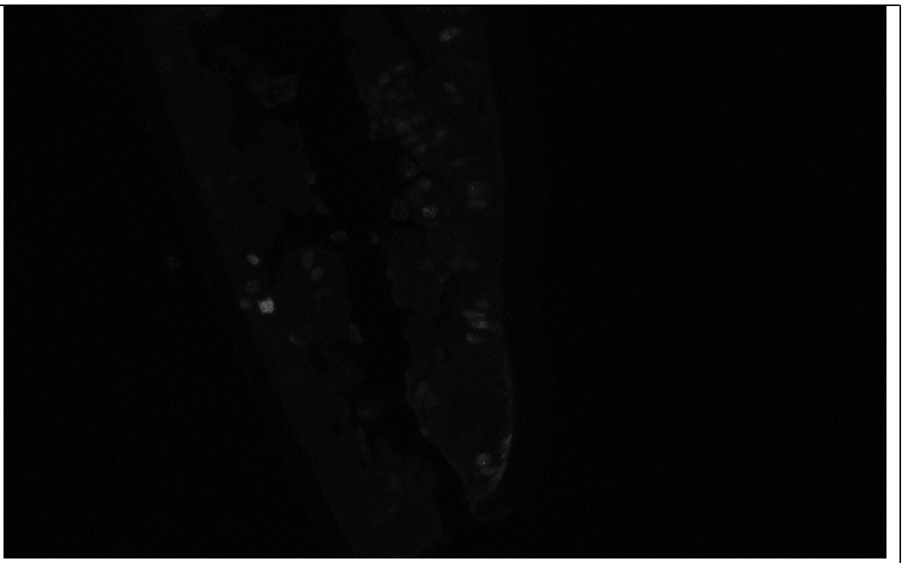
Calu-3 BMGLS 0.3%<br>Cy5	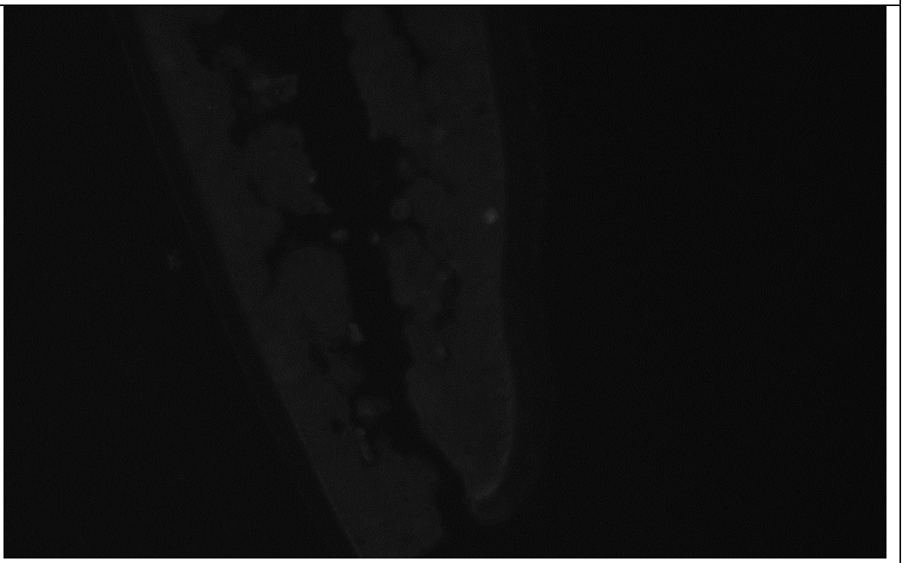
Calu-3 BMGLS 0.3%<br>DAPI	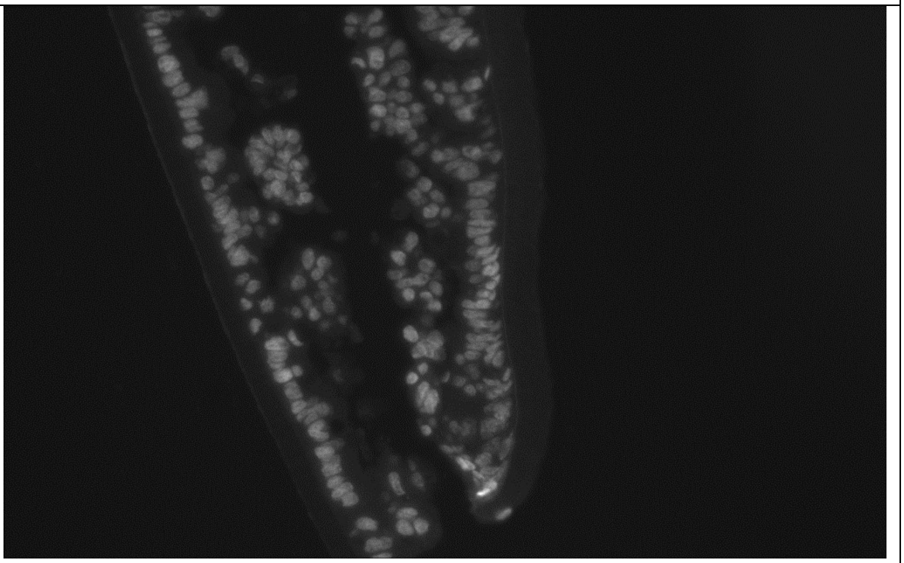

Calu-3 BMGLS 0.6%<br>Cy3	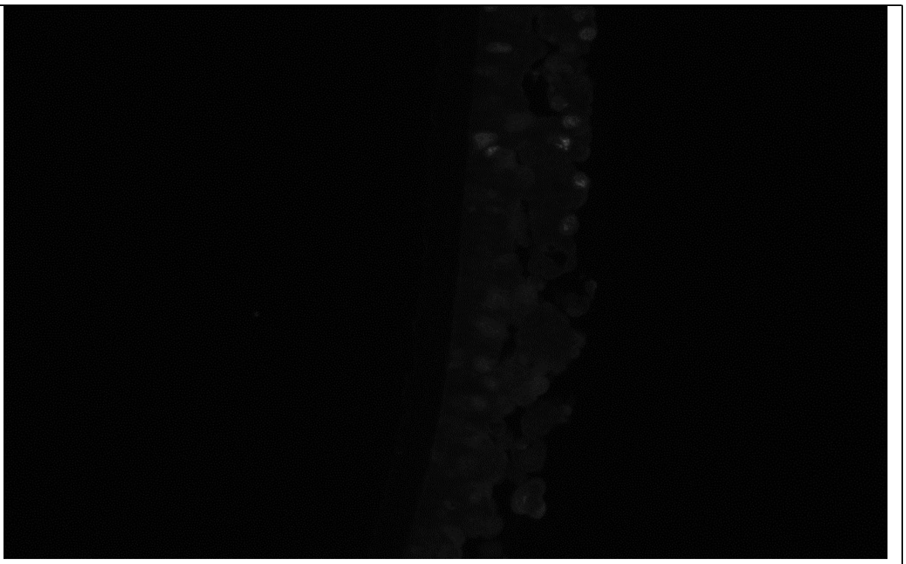
Calu-3 BMGLS 0.6%<br>Cy5	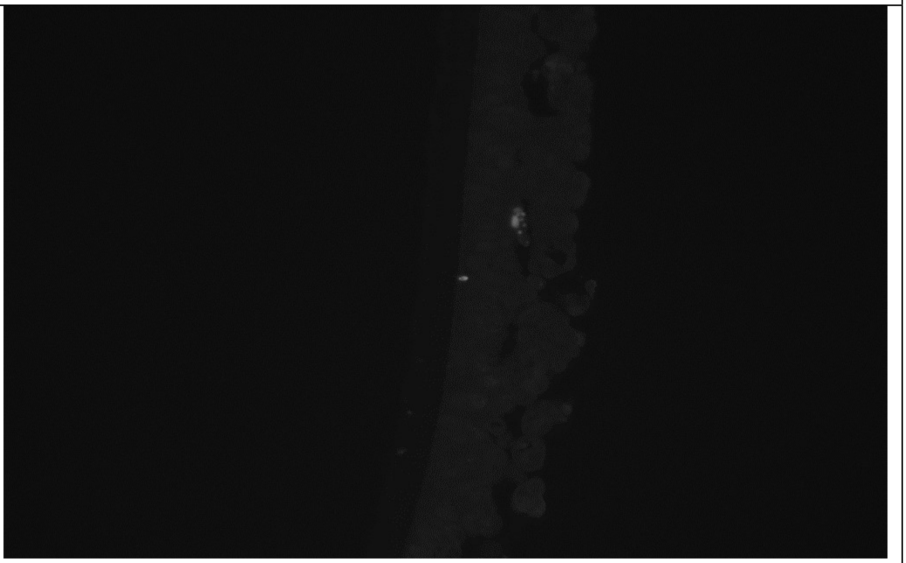
Calu-3 BMGLS 0.6%<br>DAPI	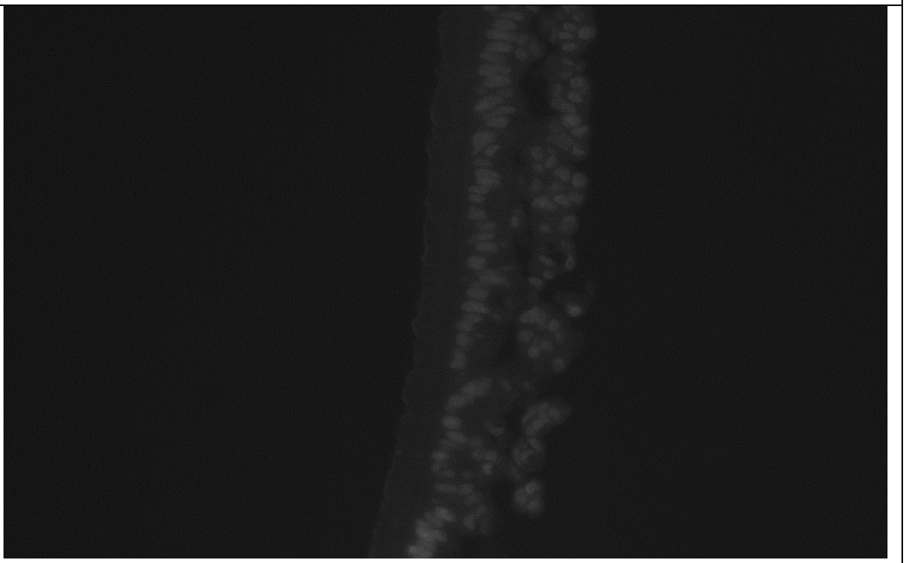

Representative IF images of BMGLS treated NHBE ALI cultures

Analysis of BMGLS treated NHBE ALI cultures. Calu-3 ALI cultures were treated with different concentrations of BMGLS over the course of 3 weeks. Representative IF image of proliferating cells (Ki67, Cy3), apoptotic cells (cPARP, Cy5) and nuclei (DAPI). Magnification 20x.

NHBE Cntrl Cy3	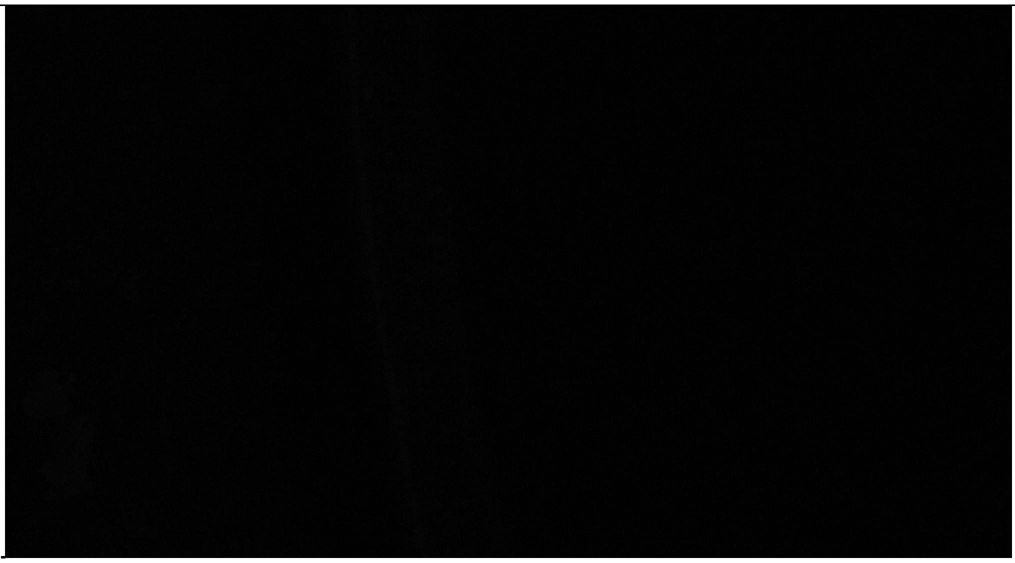
NHBE Cntrl Cy5	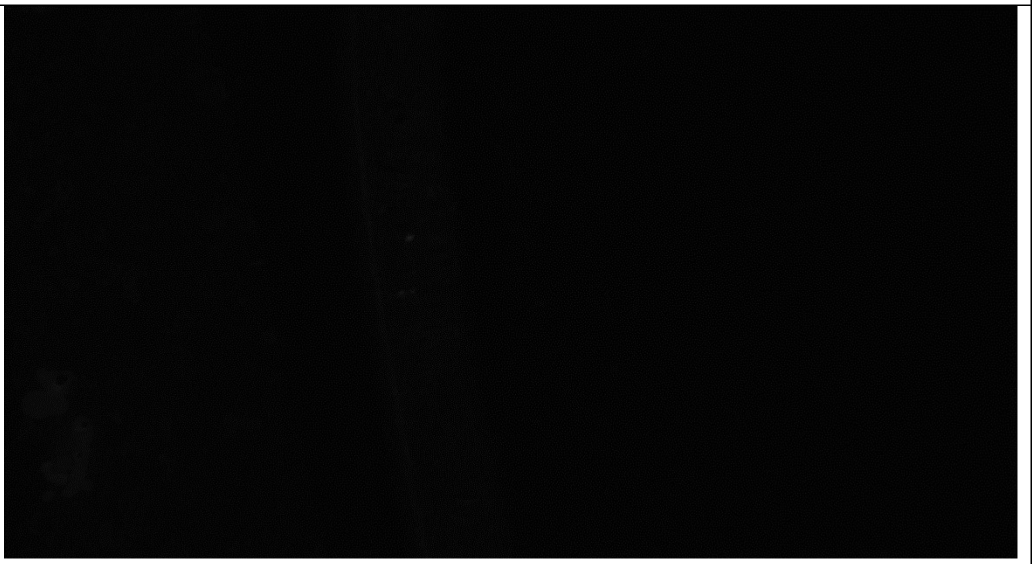

NHBE Cntrl<br>DAPI	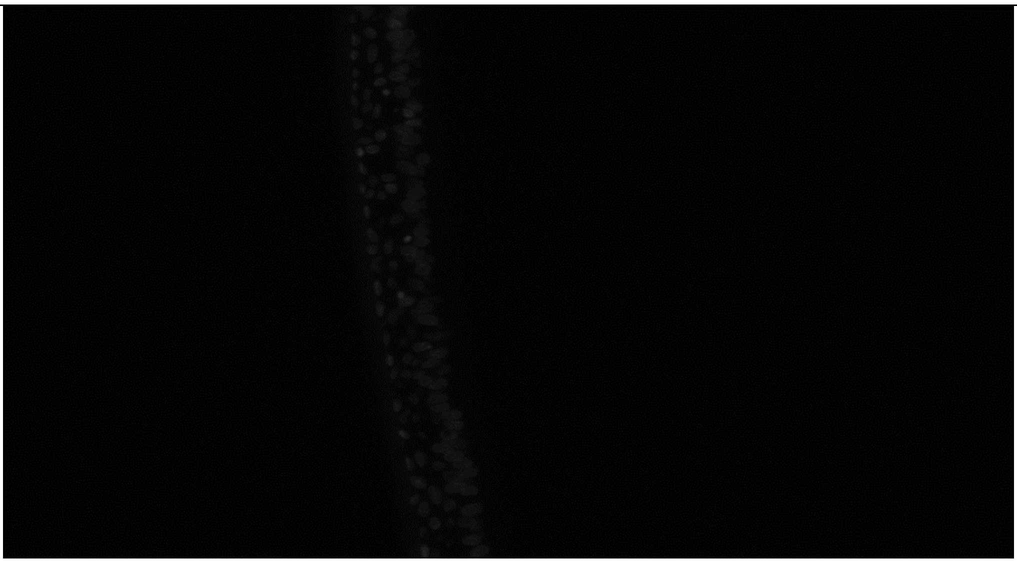

NHBE HCS<br>Cy3	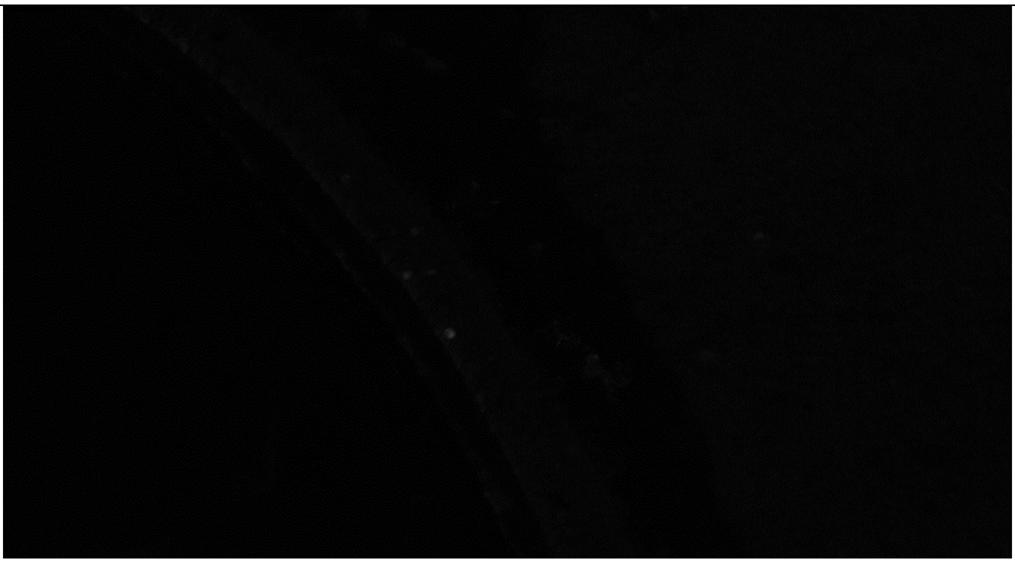
NHBE HCS<br>Cy5	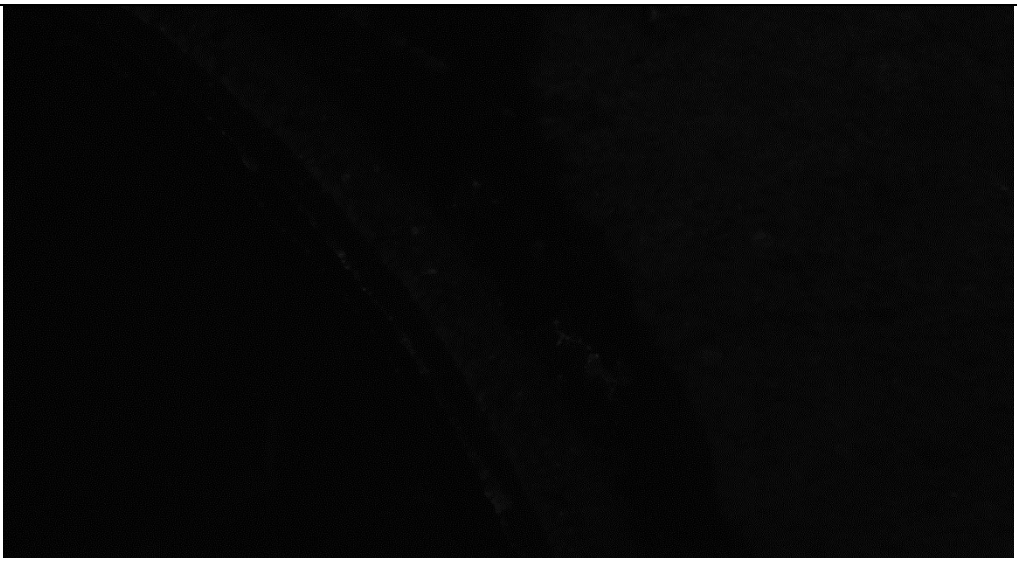

NHBE HCS<br>DAPI	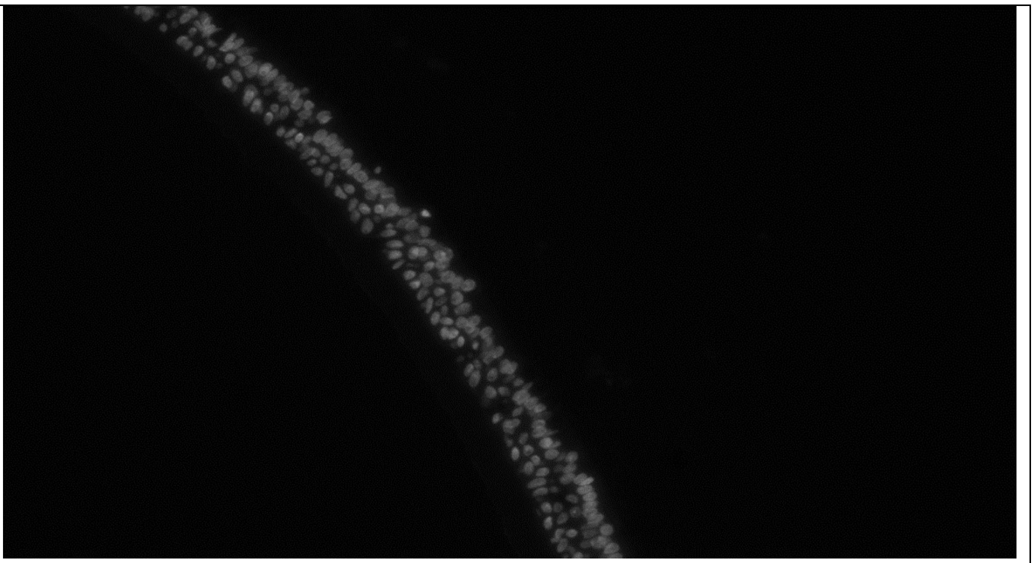

NHBE BMGLS<br>0.15% Cy3	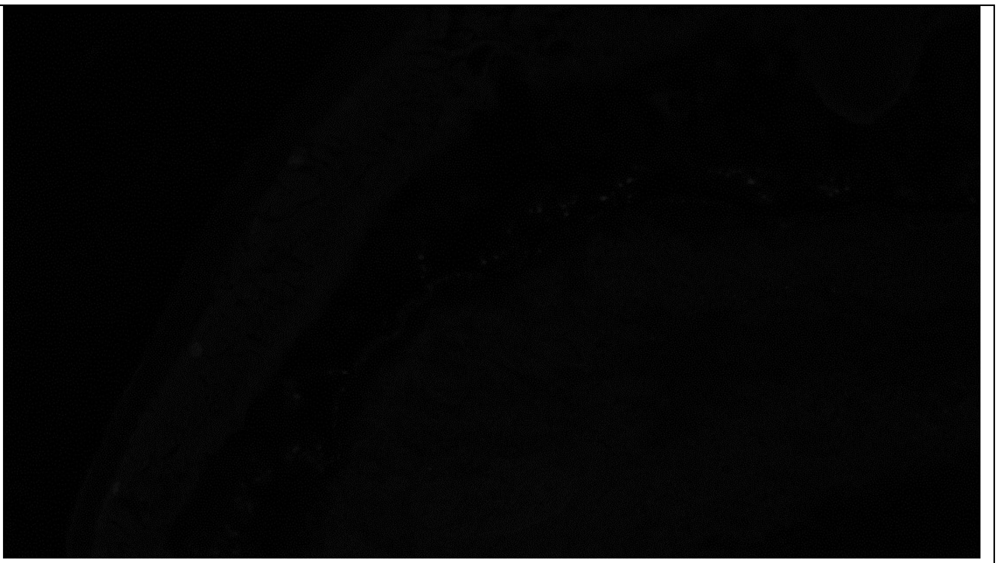

NHBE BMGLS<br>0.15% Cy5	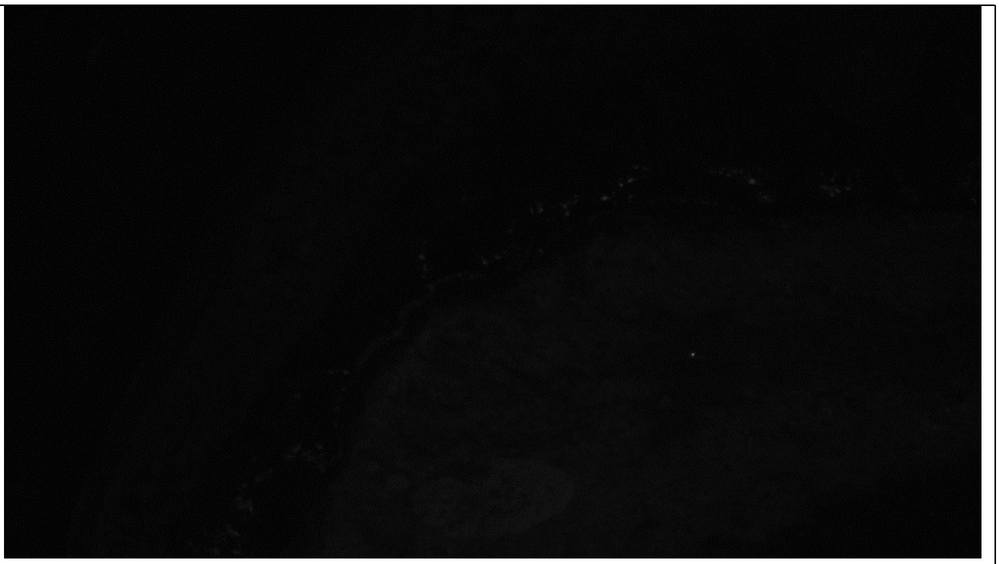
NHBE BMGLS<br>0.15% DAPI	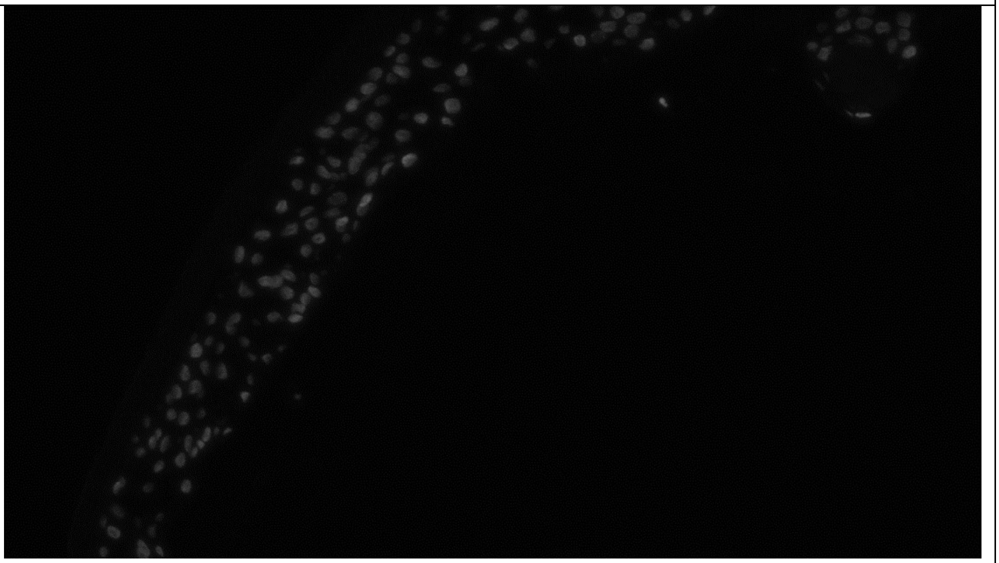

NHBE<br>BMGLS<br>0.3% Cy3	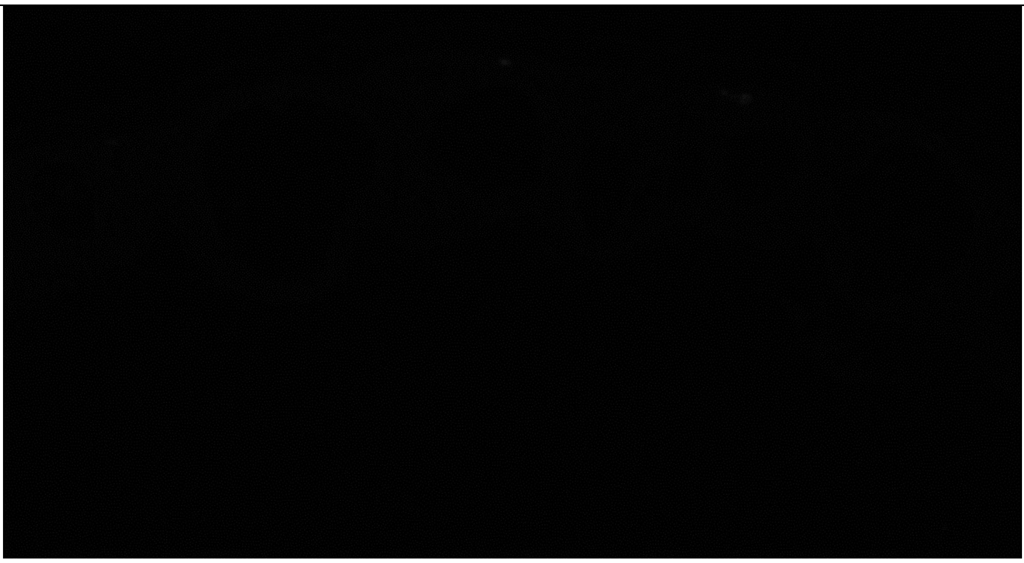
NHBE<br>BMGLS<br>0.3% Cy5	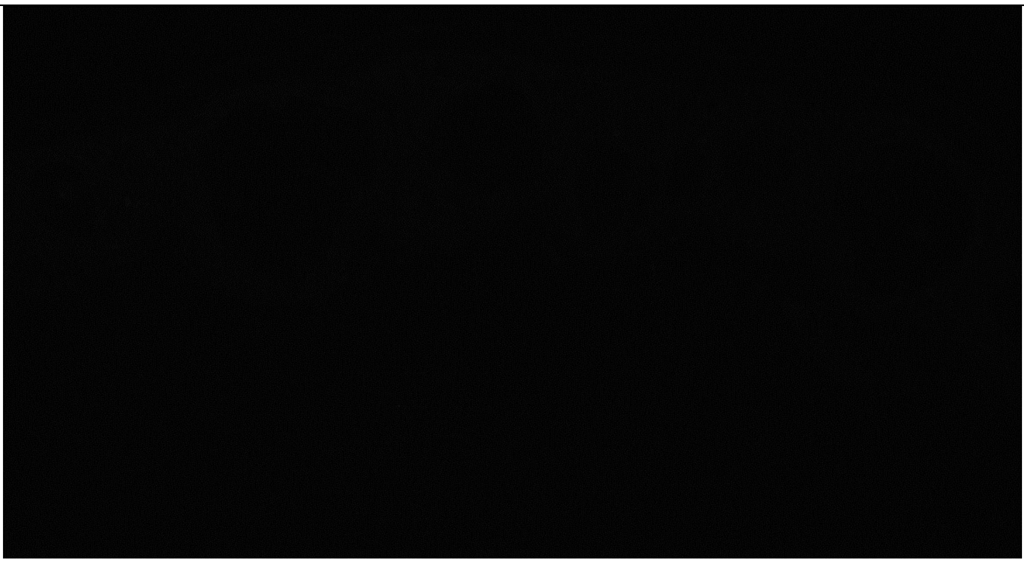
NHBE<br>BMGLS<br>0.3% DAPI	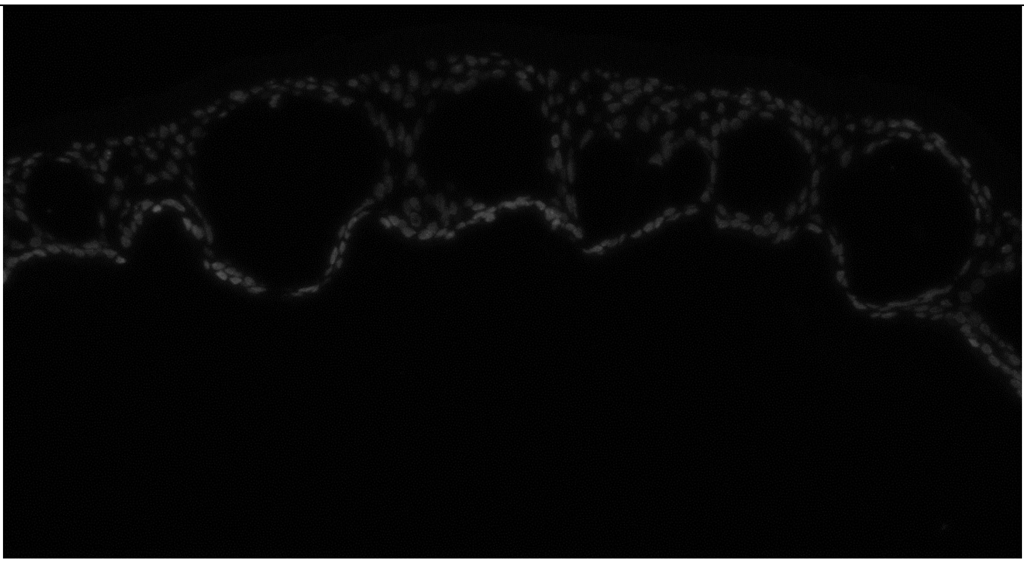

NHBE<br>BMGLS 0.6%<br>Cy3	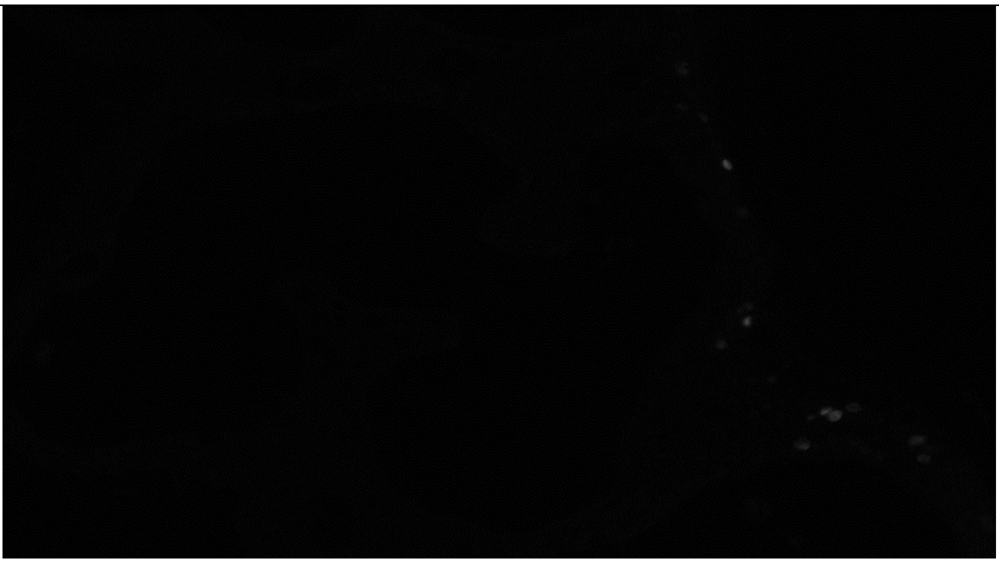
NHBE<br>BMGLS 0.6%<br>Cy5	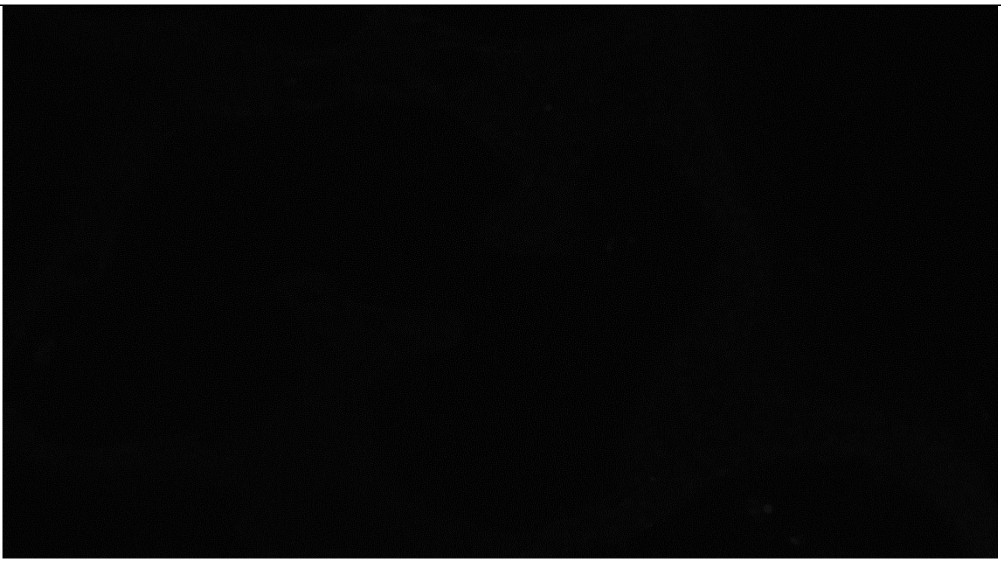
NHBE<br>BMGLS 0.6%<br>DAPI	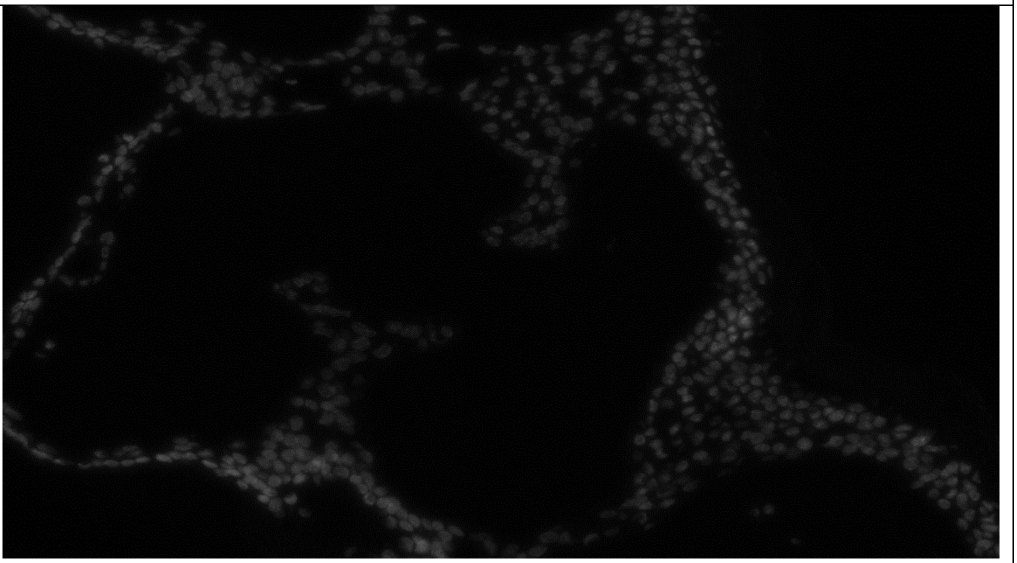
